# Supplementary material for: The interactome of intact mitochondria by cross-linking mass spectrometry provides evidence for coexisting respiratory supercomplexes
Source: Mol Cell Proteomics. 2017 Dec 8;17(2):216–32. doi: 10.1074/mcp.RA117.000470 (PMC5795388; doi:10.1074/mcp.RA117.000470)
Supplement: Supplemental Data [file supp_RA117.000470_133922_0_supp_23978_fzffwf.zip › spectra_annotation/mito_DR_spectra_annotation/51-1-87-1-2-1.pdf]

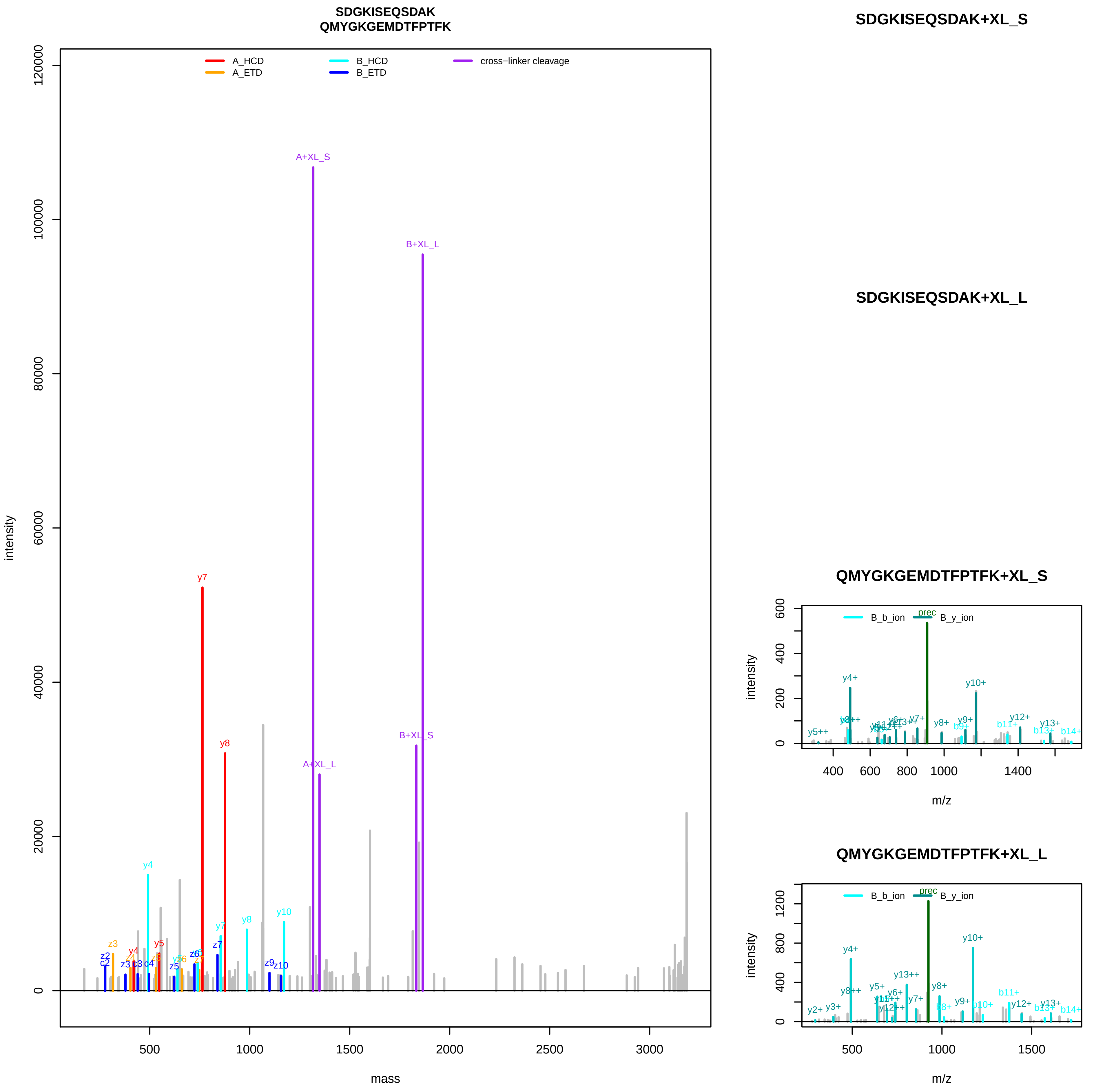
SDGKISEQSDAK
QMYGKGEMDTFPTFK
A_HCD
A_ETD
B_HCD
B_ETD
cross−linker cleavage
A+XL_S
B+XL_L
B+XL_S
A+XL_L
y7
y8
y4
y5
y10
z2
c2
z3
c3
c4
z5
z6
z7
z9
z10
intensity
mass
0
20000
40000
60000
80000
100000
120000
500
1000
1500
2000
2500
3000
SDGKISEQSDAK+XL_S
SDGKISEQSDAK+XL_L
QMYGKGEMDTFPTFK+XL_S
B_b_ion
B_y_ion
prec
y4+
y10+
y12+
y13+
y9+
y8+
y7+
b11+
b13+
b14+
y5++
m/z
400
600
800
1000
1400
0
200
400
600
QMYGKGEMDTFPTFK+XL_L
y13++
y2+
y3+
y5+
y6+
y7+
y8+
y8++
y9+
y10+
y11+
b8+
b10+
0
400
800
1200
500
1000
1500
